# Extreme Bendability of Atomically Thin MoS_2_ Grown by Chemical Vapor Deposition Assisted by Perylene-Based Promoter

**DOI:** 10.3390/nano12224050

**Published:** 2022-11-17

**Authors:** Christian Martella, Davide Campi, Pinaka Pani Tummala, Erika Kozma, Paolo Targa, Davide Codegoni, Marco Bernasconi, Alessio Lamperti, Alessandro Molle

**Affiliations:** 1 CNR IMM, Unit of Agrate Brianza, Via C. Olivetti 2, I-20864 Agrate Brianza, Italy; 2 Department of Material Science, University of Milano-Bicocca, Via R. Cozzi 55, I-20125 Milano, Italy; 3 Department of Mathematics and Physics, Università Cattolica del Sacro Cuore, Via della Garzetta 48, I-25133 Brescia, Italy; 4 Department of Physics and Astronomy, KU Leuven, Celestijnenlaan 200D, 3001 Leuven, Belgium; 5 CNR SCITEC, Unit of Milan, Via Corti 12, I-20133 Milano, Italy; 6 STMicroelectronics, Via C. Olivetti 2, I-20864 Agrate Brianza, Italy

**Keywords:** 2D materials, bending stiffness, PTAS seeding promoters, interface adhesion, strain

## Abstract

Shaping two-dimensional (2D) materials in arbitrarily complex geometries is a key to designing their unique physical properties in a controlled fashion. This is an elegant solution, taking benefit from the extreme flexibility of the 2D layers but requiring the ability to force their spatial arrangement from flat to curved geometries in a delicate balance among free-energy contributions from strain, slip-and-shear mechanisms, and adhesion to the substrate. Here, we report on a chemical vapor deposition approach, which takes advantage of the surfactant effects of organic molecules, namely the tetrapotassium salt of perylene-3,4,9,10-tetracarboxylic acid (PTAS), to conformally grow atomically thin layers of molybdenum disulphide (MoS_2_) on arbitrarily nanopatterned substrates. Using atomically resolved transmission electron microscope images and density functional theory calculations, we show that the most energetically favorable condition for the MoS_2_ layers consists of its adaptation to the local curvature of the patterned substrate through a shear-and-slip mechanism rather than strain accumulation. This conclusion also reveals that the perylene-based molecules have a role in promoting the adhesion of the layers onto the substrate, no matter the local-scale geometry.

## 1. Introduction

Two-dimensional (2D) materials display an exceptional ability to bend at high-curvature angles without rupture. This superior mechanical property provides the condition to explore the transformation into arbitrarily curved geometries as a manufacturing paradigm towards material engineering by design at the nanoscale or even at the atomic scale [1,2]. Shape transformations have been proven to impact the optical spectral features of 2D materials by modulating the nature and amplitude of the optical bandgap [3]. For instance, inducing a controlled curvature in 2D layers at the micro- and nano-scale is an effective way to spatially confine the photoluminescence emission, e.g., by the exciton-funneling effect, with potential technological implications in the creation of deterministic single-photon quantum emitters and excitonic devices [4,5,6]. In addition, theoretical calculations have predicted that simple shape transformations, involving uniaxial strain, could induce dramatic modifications in the electronic band structure in transition-metal dichalcogenides (TMDs) and monoelemental Xenes [7,8,9]. As a consequence of these modifications, an increase in the carrier mobility is expected to occur in the 2D material because of the lift in the electronic valley degeneracy and the sizeable shift in the valley energy position, ending up in the effective suppression of the intervalley electron–phonon interaction [7,8,9]. This mechanism is the basis of the mobility boosting observed in field effect transistors (FETs) based on 2D MoS_2_ layers, morphologically engineered through the deposition on a surface with a high but locally uncontrolled corrugation [10].

In MoS_2_, and TMDs in general, strategies to induce an out-of-plane distortion consisted of transferring atomically thin flakes on elastomeric substrates or on pre-patterned substrates [11]. In the former case, the shape transformation of the planes is obtained by applying an external (compressive or tensile) stimulus [12,13,14], in the latter one, by taking advantage of the intrinsic bendability in the layers to replicate the superficial topographical modulations of the substrate [15,16]. On the other hand, aiming at a deterministic control of the local curvature, the direct growth of curved MoS_2_ layers has been performed via sulfurization of molybdenum thin films pre-deposited on patterned substrates in a heterogenous (solid-vapor) chemical vapor deposition (CVD) approach [17]. Conformality of the growth, namely the condition where the ratio between the curvature angles of the MoS_2_ layers and that of the substrate pattern modulation is close to unity, is demonstrated in the so-obtained polycrystalline material up to curvature angles of ~50° [18]. However, the observed crystal order is limited to a single-grain-length scale, whose typical size is in the order of tens of nanometers [17]. The extension of the crystal-order-length scale, while keeping the conformality condition, requires a pure CVD approach where both precursors react in the vapor phase to avoid nanoscale polycrystallinity [19,20].

To date, the CVD approach is considered to be one of the best candidates to obtain large-area and high-quality TMD growth (and MoS_2_ in particular) on a variety of technologically relevant substrates [21,22,23]. Moreover, it allows for a fine tuning of the structural and morphological properties of the material by acting on the thermodynamic quantities and configurational details of the growth (including deposition temperature, pressure, carrier gas fluxes, etc.) [24]. As a matter of fact, the CVD deposition is driven by mechanisms at the substrate surface that involve atom diffusion, wettability, and the interfacial energy barrier between the 2D layers and the substrate. From theoretical and experimental perspectives [25,26], the use of seeding promoter molecules is known to be beneficial for obtaining the large-scale growth of MoS_2_ layers on dielectric flat substrates. More in detail, atomistic simulations revealed that the involved mechanisms may depend on the polar groups of the aromatic seedings acting as preferential sites for S atom adsorption [26], while experimental evidence points to the role of the perylene core that remains intact during the typical high-temperature deposition of MoS_2_ [20].

In this work, we aim at showing that aromatic perylene-based molecules, namely the tetrapotassium salt of perylene-3,4,9,10-tetracarboxylic acid (PTAS), harness adhesion and bendability of atomically thin MoS_2_ layers, even in proximity of sharp edges or extreme curvature of the dielectric substrate surface. From the interplay of CVD growth and arbitrarily patterned SiO_2_ substrates, we show that conformality is achieved at extremely sharp pattern edges only by taking benefit from the surfactant effect of the seeding promoter functional groups. Based on atomically resolved TEM images and energetic considerations, within a density functional theory (DFT) framework, we conclude that the so-grown MoS_2_ multilayers adapt to the local curvature of the patterned substrate, adopting the shear-and-slip mechanism rather than strain accumulation. From this outcome, we also infer that the role of the PTAS molecules is to retain a nearly constant adhesion energy, no matter the substrate geometry in use (ranging from flat surface to extreme geometric features, such as sharp asperities, edges, or curvatures).

## 2. Materials and Methods

*Substrate preparation*: The nanoscale patterns were fabricated in a SiO_2_ substrate by reactive ion etching through a photoresist mask. Before loading the SiO_2_ (90 nm)/Si^++^ substrates in the CVD apparatus, they were cleaned in acetone (for 5 min) and isopropanol (for 5 min). Subsequently, the substrates were washed with deionized (DI) water for 5 min and dried by means of a nitrogen flux. We used a solution of seeding promoter, namely the tetrapotassium salt of perylene-3,4,9,10-tetracarboxylic acid (PTAS) in DI water, to condition the SiO_2_ substrate. We used 0.58 mg of PTAS mixed in 10 mL distilled water to obtain a 100 µM/L solution. The solution was spread on the substrate (surface = 2 cm^2^) by delivering 10 drops of solution (~50 mg) using a pipette, thus, resulting in an approximate 0.015 mg of PTAS/cm^2^. The substrate was kept at 90 °C on a hot plate for solvent evaporation.

*MoS_2_ deposition*: MoS_2_ growth was carried out using atmospheric-pressure chemical vapor deposition using powder precursors of sulfur (99.98%, Merck KGaA, Darmstadt, Germany) and molybdenum trioxide (MoO_3,_ 99.97%, Sigma-Aldrich). The growth procedure takes place in a two-zone furnace apparatus (planarTECH LLC, Cambridge, UK) with 2” quartz tube. Before starting the CVD process, the system was pumped down to a pressure of 3 × 10^−4^ mbar then purged with 1000 sccm high-purity argon for several minutes. The MoS_2_ precursors were placed in quartz boats, sulfur in the upstream region and MoO_3_ in the downstream region. See Supplementary Materials (Figure S1) for additional details.

*Scanning Electron Microscope characterization (SEM)*: The morphology of the samples was examined using a Zeiss-SUPRA 40 field-emission SEM device (Oberkochen, Germany) in bright-field mode.

*Lamellae preparation*: The samples were prepared by means of Focused Ion Beam (FIB). The lamellae preparation was performed using a Thermo-Fisher Helios G4 FIB (Thermo-Fisher Scientific, Waltham, MA, USA). In all the cases, particular care was taken to limit heating and ballistic effects of ion irradiation on MoS_2_ film during the final ion milling steps.

*Transmission Electron Microscopy characterization*: The lamellae were investigated by means of Scanning Transmission Electron Microscopy (STEM) techniques. The images were performed with a Thermo-Fisher Themis Z G3 (Thermo-Fisher Scientific, Waltham, MA, USA) aberration-corrected transmission electron microscope equipped with an electron gun monochromator operating at 200 kV acceleration voltage.

*DFT calculations*: The smaller nanotube with a radius of 26.27 Å close to the experimentally observed one can be realized by folding 30 unit/cells and allows for the realization of the outer layer consisting of 37 unit/cells in a minimal-strain configuration where only a 0.03% expansion is needed to obtain a structure in which the van-der Waals gap has the same size as in the bulk. The bending energy (associated with the deformation of the outer and inner Mo-S bonds within the trilayer) was computed by comparing the energy of a flat MoS_2_ sheet modeled by a simulation cell with periodic boundary conditions made of replicas of a non-unitary rectangular cell along the zigzag direction with the energy of a nanotube built from the same cell. The slipping energy was computed using 30-unit (inner)–37-unit (outer) double-wall nanotube, keeping fixed the inner nanotube and rotating the outer one. Finally, the global strain energy was computed in the fixed-ion approximation by using a single-wall 30-unit nanotube and globally expanding its average radius. All the calculations were performed using the CP2k code [27] with a tripleZ-Gaussian basis set and a cutoff of 240 Ha on the plane wave expansion of charge density. We used the Perdew–Burke–Ernzherof (PBE) [28] approximation for the exchange-correlation functional and the semiempirical Grimme-D2 [29] correction to take into account the long-range van der Waals interactions. A vacuum space of 30 Å was used to decouple the nanotube from the periodic replica.

## 3. Results

**Figure 1:**
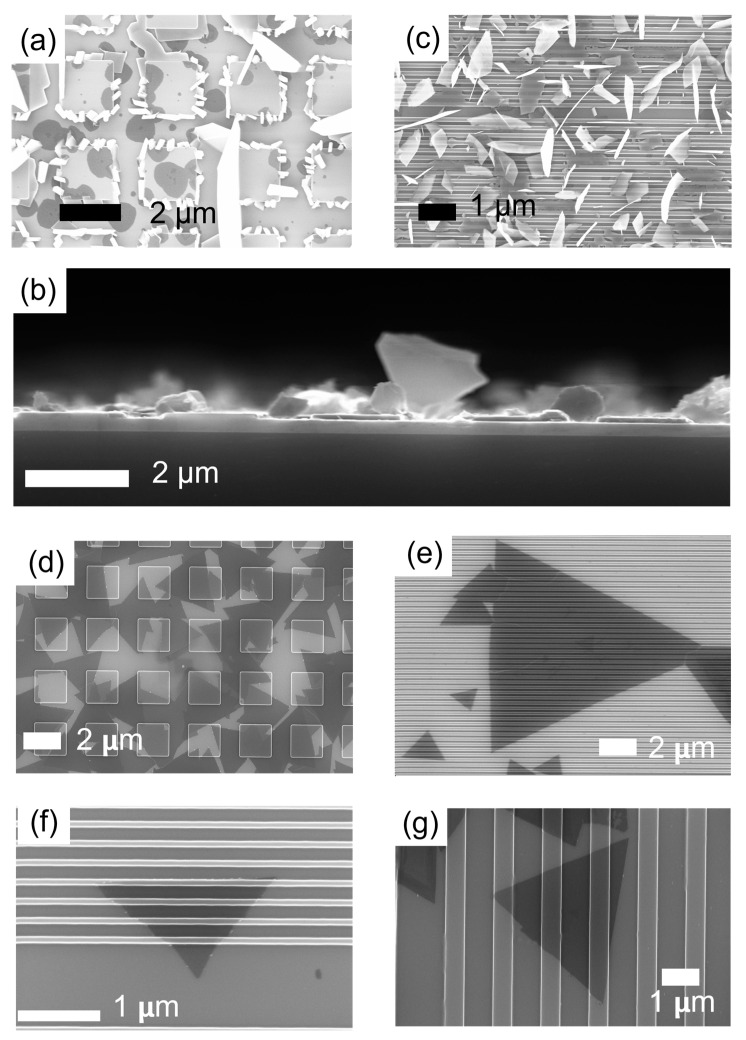
(**a**–**c**) Scanning electron micrographs showing the crystal morphologies for MoS_2_ platelets formed on patterned SiO_2_ substrates without the use of seeding promoters. In (**b**) an SEM cross-sectional view of the platelets is shown. (**d**–**g**) Scanning electron micrographs showing the in-plane morphologies of two-dimensional MoS_2_ crystals formed on patterned SiO_2_ substrates using perylene-based molecules as seeding promoters for the growth.

The growth of MoS_2_ layers on the patterned substrates presents stark differences when carried out with or without the seeding promoters. In the absence of the perylene-based molecules, the MoS_2_ layers preferentially grow with the basal planes mostly aligned with the normal direction with respect to the substrate surface, while they lie flat on the surface when the promoters are used. Inspecting the SEM images shown in Figure 1a–c, one can notice that, in the absence of the aromatic seedings, the MoS_2_ formation results in sub-micrometer crystallites grown along the sides of the pattern features, suggesting that they act as centers for the heterogeneous nucleation of the MoS_2_ clusters, Figure 1a.

**Figure 2:**
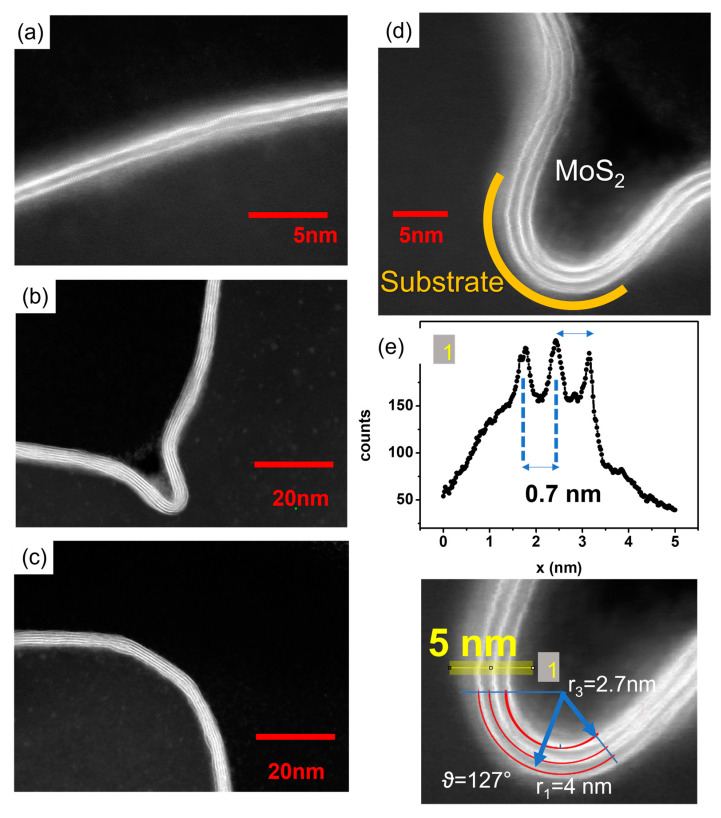
HAADF-STEM images. (**a**–**d**) cross-sectional HAADF-STEM images showing the MoS_2_ layers adapting to the substrate curvature even at the highest values. In HAAD-STEM imaging of the MoS_2_ layers, the bright contrast is obtained at the molybdenum atoms due to the higher atomic mass compared to sulphur atoms. (**e**) Intensity line profile along the yellow path (indicated with “1”) in the zoomed-in figure of panel (**d**). The peaks in the plot indicate the position of the molybdenum atoms in the MoS_2_ layers.

In addition, bigger platelets with an out-of-plane orientation can be observed to be randomly distributed on the surface, see cross-section SEM image in Figure 1b. Similarly, according to what was observed at the edges of flat substrates, we can rationalize that the out-of-plane growth of the MoS_2_ cluster in proximity to the surface pattern features occurs as a consequence of a concentration gradient component, with direction normal to the substrate through the Mullins–Sekerka mechanism [30]. Platelet formation is more evident in the case of the trench pattern shown in Figure 1c, where it is worth noting that the uniaxial orientation of the trenches does not drive a preferential MoS_2_ clustering, as revealed by the random spatial alignment of the platelets on the substrate surface. On the contrary, the use of the PTAS molecules hinders platelet and cluster formation by promoting the wettability of the dielectric SiO_2_ surface, even in the case of the patterned substrates. In this respect, Figure 1d–g show a gallery of SEM images, confirming the deposition of large-area 2D MoS_2_ crystals reproducing the spatial modulations of a variety of patterned substrate morphologies, see also Figures S2 and S3 in Supplementary Materials. As a matter of fact, large coverage of the surface takes place as a consequence of the in-plane lateral merging of the characteristic triangular single-crystal MoS_2_ domains, see Figure 1d. These findings suggest that the PTAS molecules help to promote the formation of the layers at the SiO_2_ interface in a competition between adhesion and bending. To get a closer insight into this aspect, cross-sectional images along the crystalline armchair direction were acquired using an aberration-corrected scanning transmission electron microscopy (STEM) in few-layer crystal domains accommodated on the trench-patterned substrate. Moreover, we used high-angle annular dark-field (HAADF)-STEM to resolve the atomic arrangements at specific locations of interest where the layer curvature reaches the highest values, Figure 2a–d. The HAADF-STEM images capture the stark difference in the atomic number (Z) of the Mo and S elements, Z = 42 and 16 for molybdenum and sulphur, respectively. Indeed, in HAADF imaging, the scattering cross-section of electrons follows a Z^2^ law (pure Rutherford scattering) [31]; thus, in Figure 2d, the “heavier” molybdenum atoms appear brighter than sulphur atoms. With this indication, one can observe, in Figure 2d,e, three conformal layers of MoS_2_ grown on the patterned substrate surface.

**Figure 3:**
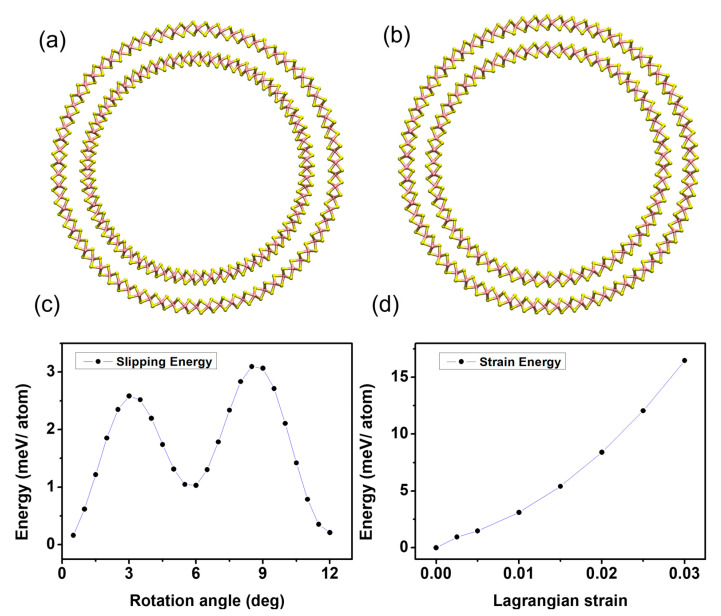
(**a**) Double-walled zig-zag nanotube used to model the high-curvature regions in a hypothetical strained configuration preserving the bulk A-B-C arrangement for both the internal and external layer made of 30-unit cells. (**b**) Double-walled nanotube used to model the high-curvature in an unstrained configuration, breaking the registry between the inner and outer layer. The inner wall counts 30 unit cells while the outer one 37. (**c**) Slipping energy obtained by rotating the inner wall of the model described in (**b**) keeping the outer wall fixed. (**d**) Strain energy computed by expanding the isolated 30-unit-cell inner nanotube.

In 2D-layered materials, such as MoS_2_, an accurate energetic description of the flat-to-curved geometry transformations relies on a delicate balance between several factors. To this scope, from STEM pictures, we argue that the growth occurs without delamination from the substrate; therefore, it is reasonable to assume that the layers are in thermodynamic equilibrium. Most noticeably, within the single S-Mo-S layer, the curvature necessarily breaks the symmetry between the upper (the further apart from the curvature center) and lower Mo-S bonds, forcing the first one to be longer and the latter one to be shorter than in the undeformed flat case, even if the average Mo-Mo distance is kept fixed. In the presence of multiple layers, additional contributions to the energy balance might rise from the inter-layer interactions. In a curved geometry, in fact, subsequent MoS_2_ layers with a different distance from the curvature center could either try to retain an AB stacking, corresponding to the most favorable configuration in DFT for flat isolated bilayers [32], accommodating the different curvature as an overall strain of the layer (a uniform change in Mo-Mo and Mo-S bond lengths), or move away from the bulk configuration in favor of a disproportionate stacking through slip-and-shear mechanisms of the individual single layers [33,34]. Furthermore, the adhesion energy between the layers might change as a result of the distorted bond geometry of the layers facing the van der Waals gap or a modulation of the inter-layer distance. A simple energy balance equation at the high-curvature points or edges of the surface modulation must, thus, take into account the competition among four different energetic contributions, namely adhesion with the substrate, bending, strain, and inter-layer binding energy as effective contributions to the overall surface free-energy balance [33,34]. In order to gain a deeper insight into the relative importance of the aforementioned energy terms and the structural behavior of the MoS_2_ multilayer in strongly bent configurations, we performed DFT calculations, in which we modelled the high-curvature region of our system (Figure 2d) as coaxial double-wall zig-zag nanotubes with curvatures similar to the experimentally observed ones and separated by the same van der Waals distance observed in bulk. As a first step, we built two different systems. In the first one, the inner layer is globally strained in order to maintain the ideal bulk registry with the outer one, the outer one is strain free (the radius has been chosen in order to obtain a circumference that matches the length of 37 unit cells in their equilibrium geometry and no sensible variation of the radius has been observed letting the system free to relax), and the positions of the Mo atoms are kept fixed to mimic the effect of the substrate, Figure 3a. In the second case, instead, both the inner and outer layers are in a nearly strain-free configuration (only the inner one is subject to a 0.03% compression in order to commensurate the structure and keep the same inter-layer distance observed in bulk), the length difference between the two is compensated by removing unit cells in the inner layer, inevitably breaking the registry with the outer one, Figure 3b.

Despite the first model releasing a considerable amount of strain with a 0.6 Å reduction in the inter-layer distance, the second configuration turned out to be energetically favorable, with an energy difference of 262 meV/atom. To give a more detailed estimate of the energy terms at stake, we computed, for the most favorable model, the energies associated with the bending, inter-layer interaction, the breaking of lattice bulk stacking (slipping energy), and the possible overall strain in the curved configuration. The results are reported in Figure 3c,d, respectively. The computational procedure for each quantity is detailed in the Materials and Methods section. As a result, it turns out that the observed conformality of the MoS_2_ nanosheet grown by PTAS-assisted CVD is consistently rationalized by a model where the MoS_2_ layers are minimally affected by atomic strain, corresponding to extremely sharp asperities of the substrates. It is, then, interesting to elucidate what is the key mechanism governing this apparent slippery in the MoS_2_ nanosheets, no matter the substrate corrugations. Starting from this point, in the next section, we attempt to rationalize the experimental picture in terms of the surface and interface free-energy contributions coming into play in the nanosheet growth and so reach a full understanding of how to bend the MoS_2_ nanosheets by design.

## 4. Discussion

Notably, according to our DFT calculations, the slipping energy barrier is predicted to be fairly small (3.1 meV/atom). This fact suggests that in multilayer configurations, only a tiny amount of global strain might be energetically favorable (0.5% at most) before the system releases the strain through slipping. This observation echoes what is observed in HAADF-STEM studies of MoS_2_ and graphene of a few layers draped over atomic steps in hexagonal boron nitride substrates, where a superlubric behavior implicates the bending mechanism, in that it is primarily dominated by interlayer shear and slip rather than an accumulation of intra-layer strain [33,34]. The layer bending stiffness, ***B***, (obtained from the comparison of the isolated nanotubes with an equivalent flat monolayer) was estimated in 10.3 eV for both the internal and external nanotube, in agreement with previous calculations and experimental results [33,34]. The binding energy between the two curved MoS_2_ monolayers (obtained as the energy difference between the double-walled configuration and the two isolated nanotubes) was estimated in 0.42 J/m^2^ (that, considering our computational configuration, is ~16.3 meV/atom), surprisingly similar to the binding energy in the ideally flat configuration, despite the strongly distorted geometry. Adapting the energy-balance equation developed by Han et al. [33,34] and taking the outcome of the DFT calculation as an input in the overall energy balance, it is possible to estimate the effect of the PTAS on the MoS_2_-SiO_2_ adhesion as well as qualitatively understand the multilayer growth behavior. Since we are dealing with a layer-by-layer growth rather than a multilayer deposition on a corrugated surface, it is useful to write the energy balance as

(1) $$E=\overset{N}{\underset{i=1}{\sum }}\Gamma_{i}L_{i}+B_{i}\theta_{i}/R_{i}$$

where the index ***i*** runs on the number of layers, ***E*** is the total energy per unit length, $\Gamma_{i}$ represents the binding energy per surface area between a layer and the previous one (or the substrate for ***i*** = 1), $L_{i}$ the contact length, $\theta_{i}$ the angle of constant curvature and $R_{i}$ the curvature radius, ***B_i_*** the layer bending stiffness, and we neglected the strain contributions. Considering the first layer and noting that, in our geometry, we have a perfect adhesion over the entire circumference arc (namely ***L*** = θR), the proposed model predicts that the interfacial adhesion energy, ***Γ***, and the layer bending stiffness, ***B***, are in relation through the equation |***Γ***| = ***B***/***R***^2^, where R is the radius of curvature in the layers. In Figure 2d, the measured ***R***~4 nm (for the layer in contact with the substrate), by assuming ***B*** = 10.3 eV, leads to an interfacial adhesion energy of~0.1 Joule/m^2^ (~3.9 meV/atom). Notably, the derived ***Γ*** value on the patterned substrate with the use of perylene-based molecules is comparable to the reported adhesion energy of MoS_2_ layers transferred on a flat SiO_2_ substrate (***Γ*** = 0.17 J/m^2^) [35]. This quantitative similarity reflects the role of the PTAS in boosting the adhesion of the MoS_2_ to a corrugated surface, even in proximity to sharp asperities, edges, or close curvatures to a similar extent to what usually takes place in a flat, non-corrugated surface. The conformal growth of the subsequent layers is instead ensured by the MoS_2_-MoS_2_ binding energy that retains a relatively large value (0.42 J/m^2^) in the curved geometry, which is enough to compensate the extra bending energy and any additional energy term related to the shear-slip motion or small residual strain.

## 5. Conclusions

In conclusion, we have shown that single-crystal MoS_2_ layers grow conformally on the extreme asperities of pre-patterned trench-like substrates only when taking advantage of the surfactant effect of perylene-based seeding promoters. Based on atomically resolved TEM images and DFT calculations, we demonstrate that, in extreme curved geometries, the most favorable energetic configuration for the MoS_2_ multilayers is to accumulate only a tiny amount of global strain before relaxing through slipping. We also quantitatively derive the role of the perylene-based molecules in promoting the adhesion of the layers onto the substrate, no matter the local-scale geometry. To a general extent, our work opens the door to deformable 2D layers by design, whose technological potential points out the realization of flexible devices, strain sensors, quantum-photon-emitting sources and strain-engineered field-effect transistors [4,10,36].

## Data Availability

The data of this study are available in the article, supplementary materials and upon request to the Authors.

## Supplementary Materials

The following supporting information can be downloaded at: https://www.mdpi.com/article/10.3390/nano12224050/s1, Figure S1: Schematic diagram of the two-zones furnaces and thermal ramps of the CVD processes; Figure S2: Atomic force microscopy investigations of the samples, Figure S3 Raman and Photoluminescence spectroscopy investigations of the samples. References [24,37,38] are cited in the Supplementary Materials.
