# Supplementary material for: Extreme Bendability of Atomically Thin MoS2 Grown by Chemical Vapor Deposition Assisted by Perylene-Based Promoter
Source: Nanomaterials (Basel). 2022 Nov 17;12(22):4050. doi: 10.3390/nano12224050 (PMC9697825; doi:10.3390/nano12224050)
Supplement: Supplementary file 1 [file nanomaterials-12-04050-s001.zip › nanomaterials-1973715-supplementary.pdf]

nanomaterials

Supplementary Materials

Extreme Bendability of Atomically Thin MoS_2 Grown by Chemical Vapor Deposition Assisted by Perylene-Based Promoter

Christian Martella [1,*], Davide Campi [2,*], Pinaka Pani Tummala [1,3,4], Erika Kozma [5], Paolo Targa [6], Davide Codegoni [6], Marco Bernasconi [2], Alessio Lamperti [1,*] and Alessandro Molle [1,*]

1 CNR IMM, Unit of Agrate Brianza, Via C. Olivetti 2, I-20864 Agrate Brianza, Italy
2 Department of Material Science, University of Milano-Bicocca, Via R. Cozzi 55, I-20125 Milano, Italy
3 Department of Mathematics and Physics, Università Cattolica del Sacro Cuore, Via della Garzetta 48, 25133 Brescia, Italy
4 Department of Physics and Astronomy, KU Leuven, Celestijnenlaan 200D, 3001 Leuven, Belgium
5 CNR SCITEC, Unit of Milan, Via Corti 12, I-20133, Milano, Italy
6 STMicroelectronics, Via C. Olivetti 2, I-20864 Agrate Brianza, Italy
* Correspondence: christian.martella@mdm.imm.cnr.it (C.M.); davide.campi@unimib.it (D.C.); alessio.lamperti@mdm.imm.cnr.it (A.L.); alessandro.molle@mdm.imm.cnr.it (A.M.)

1. Experimental details of the MoS_2 growth

MoS_2 layers reported in this work has been grown chemical vapor deposition (CVD) using sulfur powder (S, 99.98%, Sigma-Aldrich) and molybdenum trioxide powder (MoO_3, 99.97%, Sigma-Aldrich) as precursors. Before starting the CVD process, the system was pumped down to a pressure of 3×10^{-4} mbar then purged with 1000 sccm high purity argon for several minutes. During the CVD process the pressure of the system is at atmosferic pressure.

The substrates for the growth were patterned SiO_2 (90 nm)/Si^{++} and two hotwall furnaces with 2″ quartz tube with length 150 cm where upstream(S) and downstream (MoO_3) containing boats are precisely positioned as shown in Figure S1a. During the CVD, S and MoO_3 powders with amount of 200 mg and 1 mg respectively were contained in crucibles and placed near to heating zones of upstream (furnace -1 in figure) and downstream (furnace -2 in figure) furnaces. In the CVD setup, the S and MoO_3 powders were placed in the middle of each furnaces inside of glass boats separated by 24 cm, the substrate was kept face-down above the MoO_3 containing crucible. Figure S1b shows the temperature profile adopted for the CVD growth: the maximum temperature process was 300 °C for the sulfur source and 750 °C for the MoO_3 precursors, the effective growth step last for 20 minutes. During the growth time, we introduced an Ar gas flux at 60 sccm, while it is set at 500 sccm in the other steps of the process. The temperature profile for S, with a maximum temperature of 300 °C, grants the control of the sulfur sublimation rate. For the MoO_3, a maximum temperature of 750 °C is selected on the basis of previous studies. [1]

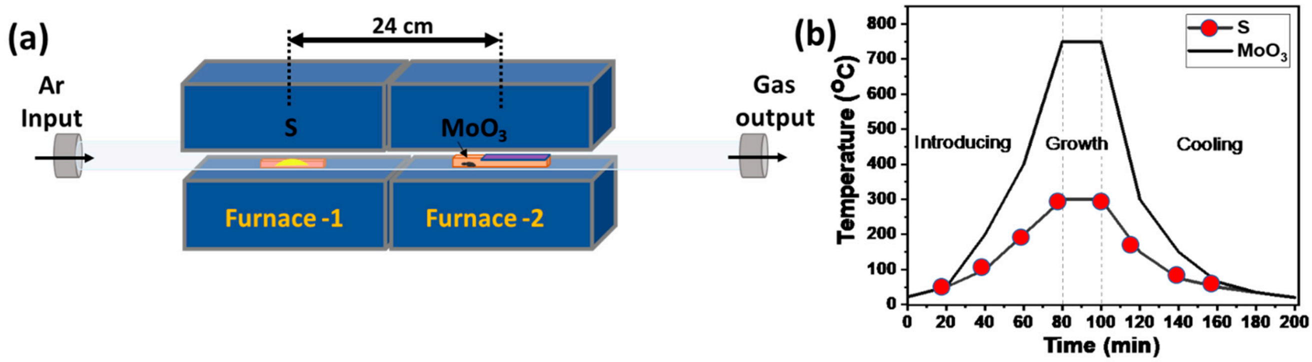

Figure S1: (**a**) Schematic diagram of the horizontal two-zones CVD furnace for the synthesis of MoS_2 layers. (**b**) Three step temperature profile (Introducing-Growth-Cooling) used for the synthesis of the MoS_2 layers with indication of the growth ramp at 750 °C for the MoO_3 and 300°C for S for 20 min.

2. Atomic force microscopy of the MoS_2 layers

Figure S2 shows the atomic force microscopy investigation of the MoS_2 layers grown on the patterned substrates. We used an AFM-Bruker commercial system (Dimension Edge) equipped with ultra-sharped silicon tips (Bruker TESPA V-2, nominal tip radius 7–10 nm) operating in tapping mode. Figure S2a) shows the 2D version of a 5 μm × 5 μm topography acquired on a selected sample area. The same topography is shown in panel b) in a 3D layout. Because of the high difference in the vertical dynamic, the 2D version of the image only shows the morphological features (trenches and ridges) of the substrate, while the presence of atomically thin MoS_2 layers with the characteristic triangular shape can be appreciated in the 3D layout (white arrows). The phase contrast image shown in Figure S2c) allow us to more clearly identify the MoS_2 domains that conformally adapt to the trenches (white arrows). The line profiles in panel d) and e) refer to the yellow and green paths shown in Figure S2 b, respectively. The height profile of the trenches (panel d) shows a spatial modulation of period ~ 250 nm (peak-to-peak distance) and vertical amplitude of ~ 35 nm. The height profile of panel e) shows a thickness of ~ 2.5 nm consistent with the presence of 3–4 MoS_2 layers.

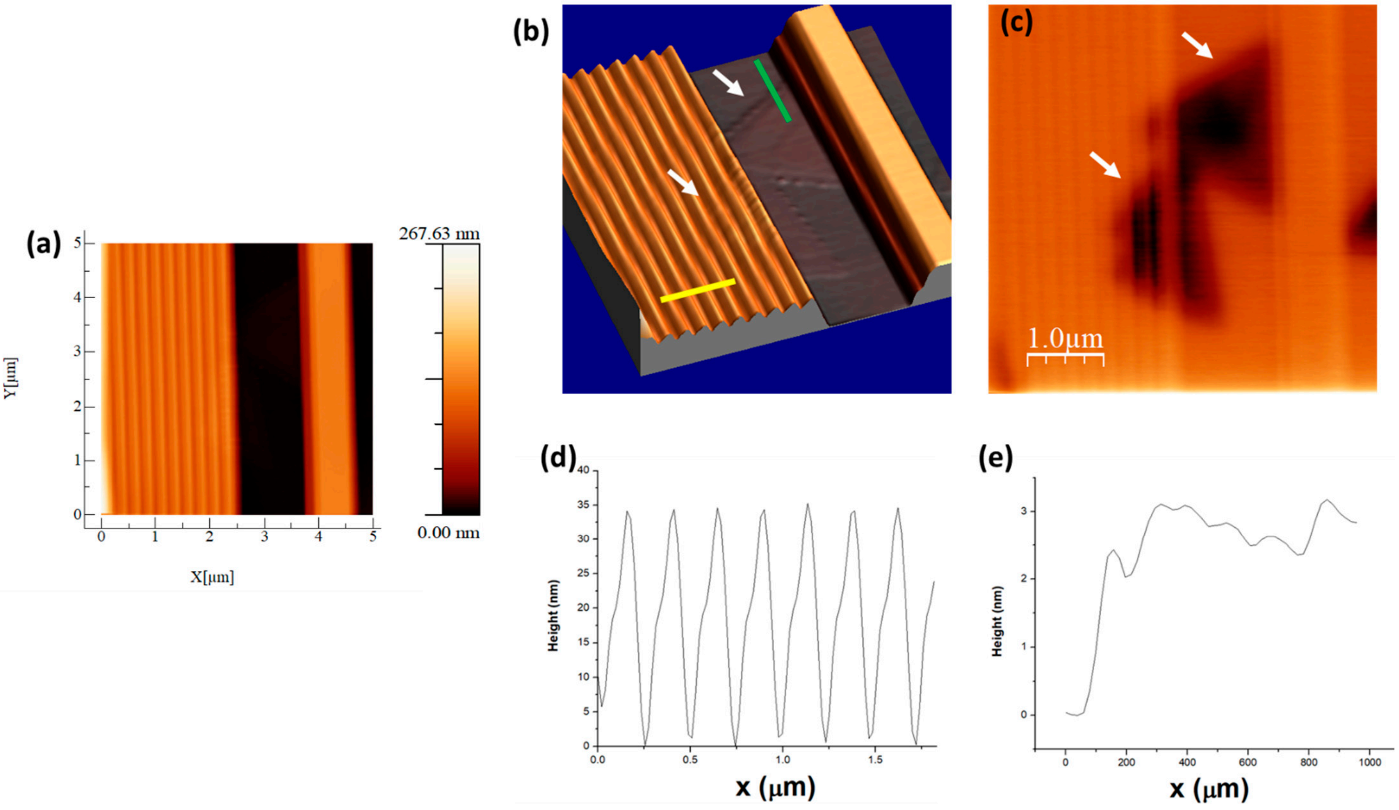

Figure S2: (**a**) 2D and (**b**) 3D AFM topographies of the MoS_2 layers grown on the patterned SiO_2/Si substrate. (**c**) phase contrast of the topography in (**a**). (**d**) and (**e**) height profile along the yellow and green paths shown in panel b, respectively.

3. Raman and photoluminescence spectroscopy of the MoS_2 layers

Micro-Raman and photoluminescence (PL) spectroscopy was carried out by means of a Renishaw In-Via spectrometer (New Mills, Kingswood, Wotton-under-Edge, UK) equipped with a solid-state laser source (wavelength 514 nm) in backscattering configuration. The laser beam was focalized through a 50x magnification objective (numerical aperture 0.75) and, to avoid sample damage, the incident power was kept below 1 mW. Figure S3a shows the characteristic Raman spectrum from MoS_2 layers grown on the patterned SiO_2/Si substrate, see SEM image in Figure. The spectrum presents the E^1_{2g}(in-plane) and A_{1g} (out of plane) phonon modes located at ~382 cm^{-1} and 406 cm^{-1}, respectively. The spectral separation of the two modes of 24 cm^{-1} is compatible with the formation of 3–4 MoS_2 layers.[2] Figure S3 b represents the PL spectrum of the as-grown MoS_2 crystal. The photoluminescence emission extends over the range 1.7–1.9 eV with a full-width-at-half-maximum (FWHM) of ~0.7 eV and maximum intensity at ~1.83 eV, these values are compatible with the indirect optical transition of multilayer MoS_2 occuring at room temperature. [3]

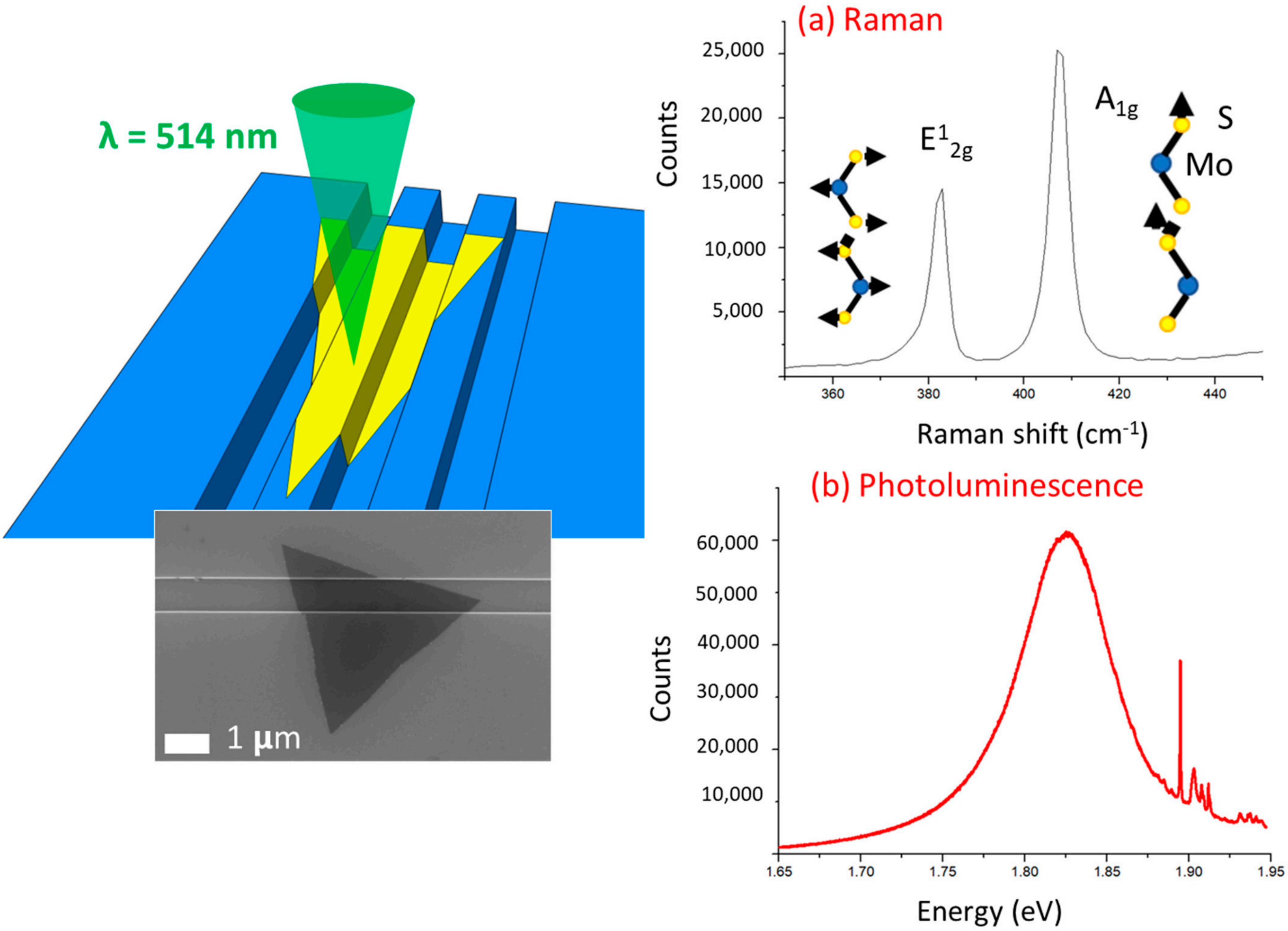

Figure S3: (**a**) Raman spectrum of the MoS_2 domain grown on the patterned substrate, see SEM image on the left. In the inset the pictorial vibrations of the S and Mo atoms are shown for the in-plane (E^1_{2g}) and out-of-plane (A_{1g}) Raman modes. (**b**) Photoluminescence emission of the same MoS_2 crystal. For both Raman and PL, the same 514 nm laser source was used as excitation radiation.

References

1. Tummala, P.; Lamperti, A.; Alia, M.; Kozma, E.; Nobili, L.G.; Molle, A. Application-Oriented Growth of a Molybdenum Disulfide (MoS_2) Single Layer by Means of Parametrically Optimized Chemical Vapor Deposition. *Materials (Basel).* **2020**, *13*, 2786, doi:10.3390/ma13122786.
2. Lee, C.; Yan, H.; Brus, L.E.; Heinz, T.F.; Hone, J.; Ryu, S. Anomalous Lattice Vibrations of Single- and Few-Layer MoS_2. *ACS Nano* **2010**, *4*, 2695–2700, doi:10.1021/nn1003937.
3. Mak, K.F.; He, K.; Lee, C.; Lee, G.H.; Hone, J.; Heinz, T.F.; Shan, J. Tightly bound trions in monolayer MoS_2. *Nat. Mater.* **2013**, *12*, 207–211, doi:10.1038/nmat3505.
